# Prevalence and risk factors for retropharyngeal and retro-styloid lymph node metastasis in hypopharyngeal carcinoma

**DOI:** 10.1186/s13014-023-02322-4

**Published:** 2023-08-11

**Authors:** Ryo Toya, Tomohiko Matsuyama, Tetsuo Saito, Yoshiyuki Fukugawa, Takahiro Watakabe, Shinya Shiraishi, Daizo Murakami, Yorihisa Orita, Toshinori Hirai, Natsuo Oya

**Affiliations:** 1 Department of Radiological Sciences, Graduate School of Biomedical Sciences, Nagasaki University, 1-7-1 Sakamoto, Nagasaki, 852-8501 Japan; 2 Department of Radiation Oncology, Faculty of Life Sciences, Kumamoto University, Kumamoto, Japan; 3 Department of Diagnostic Radiology, Faculty of Life Sciences, Kumamoto University, Kumamoto, Japan; 4 Department of Otolaryngology-Head and Neck Surgery, Faculty of Life Sciences, Kumamoto University, Kumamoto, Japan

**Keywords:** Head and neck cancer, Radiotherapy, Hypopharyngeal carcinoma, Lymph node metastasis, Retropharyngeal lymph node, Retro-styloid lymph node, Magnetic resonance imaging, Positron emission tomography, Clinical target volume

## Abstract

**Background:**

We evaluated the prevalence and identified the risk factors for retropharyngeal and retro-styloid lymph node metastasis (LNM) in patients with hypopharyngeal carcinoma (HPC). This was achieved using a combination of magnetic resonance (MR) and [^18^ F]-fluoro-2-deoxy-D-glucose (FDG)–positron emission tomography (PET)/computed tomography (CT) images.

**Methods:**

Two board-certified radiation oncologists retrospectively reviewed pretreatment FDG–PET/CT images and contrast-enhanced thin-slice CT and MR images of 155 patients with HPC who underwent radiotherapy. Fisher’s exact tests and logistic regression analyses were performed to assess the risk factors for LNM.

**Results:**

Retropharyngeal LNM (RPLNM) was confirmed in 20 (13%) patients. Posterior wall (PW) tumors (odds ratio [OR]: 4.128, 95% confidence interval [CI]: 1.339–12.727; *p* = 0.014) and bilateral or contralateral cervical LNM (OR: 11.577, 95% CI: 2.135–62.789; *p* = 0.005) were significantly correlated with RPLNM. The RPLNM was found in 9 (32%) of the 28 patients with PW tumors. Of these 9 patients, 2 (7%) had ipsilateral RPLNM, 3 (11%) had contralateral RPLNM, and 4 (14%) had bilateral RPLNM. The PW tumors were significantly associated with contralateral RPLNM (*p* < 0.001). Retro-styloid LNM (RSLNM) was confirmed in two (1%) patients, both of whom had ipsilateral RSLNM with lymph nodes (LNs) of ≥ 15 mm in the upper limit of ipsilateral level II. A significant association was found between LNs of ≥ 15 mm in the upper limit of ipsilateral level II and ipsilateral RSLNM (*p* = 0.001).

**Conclusions:**

The RPLNM was identified in 13% of patients with HPC. The PW tumors and bilateral or contralateral cervical LNM were risk factors for RPLNM; particularly, PW tumors were a specific risk factor for contralateral RPLNM. Although the RSLNM was rare, LNs of ≥ 15 mm in the upper limit of ipsilateral level II were a risk factor for ipsilateral RSLNM.

## Background

Radiotherapy (RT), with or without chemotherapy, is a standard treatment for hypopharyngeal carcinoma (HPC) performed as an organ preservation therapy [1]. The increased use of intensity-modulated radiotherapy and volumetric modulated arc therapy, which provides highly conformal dose distributions, has led to a corresponding increase in the importance of appropriate delineation and selection of target volumes [2]. In 2000, Grégoire et al. presented recommendations for the selection and delineation of lymph node (LN) levels based on computed tomography (CT) images [3], and these have been updated regularly [2, 4].

Level VII is the prevertebral compartment group and was first described in the most recent guidelines by Grégoire et al. published in 2014 [4]. This is subdivided into levels VIIa and VIIb. Level VIIa is the area extending from the upper edge of the C1 vertebral body or hard palate to the cranial edge of the body of the hyoid bone. It is delineated, medially, by the lateral edge of the longus capitis muscle; laterally, by the medial edge of the internal carotid artery; anteriorly, by the pharyngeal constrictor muscles; and posteriorly, by the longus capitis and longus colli muscles. Typically, retropharyngeal LNs (RPLNs) are divided into a medial or lateral group, with level VIIa containing the lateral group alone. Retro-styloid LN (RSLN) is located in the retro-styloid space, named as level VIIb. Level VIIb is the cranial continuation of level II, and comprises the area extending from the base of the skull to the caudal edge of the lateral process of C1. It is delineated, medially, by the medial edge of the internal carotid artery; laterally, by the styloid process and the deep parotid lobe; anteriorly, by the posterior edge of the pre-styloid para-pharyngeal space; and posteriorly, by the C1 vertebral body and the base of skull [4].

Unlike metastasis in levels I–VI, diagnosing RPLN metastasis (RPLNM) and RSLN metastasis (RSLNM) is difficult using ultrasound or clinical examination. Furthermore, data regarding the prevalence and risk factors for RPLNM and RSLNM in HPC patients are limited owing to the difficulty of surgical access and pathological data based on a small number of patients [5, 6]. Therefore, imaging modalities including CT, magnetic resonance (MR), and [^18^ F]-fluoro-2-deoxy-D-glucose (FDG)–positron emission tomography (PET), are required for the diagnosis of RPLNM and RSLNM. A combination of all three imaging modalities provides better diagnostic accuracy of RPLNM than a single imaging modality or a combination of two [7, 8]. However, previous studies that have evaluated the prevalence and risk factors for RPLNM have been based on a single modality of CT, MR, or FDG–PET [9–13]. Furthermore, to our knowledge, the prevalence and risk factors for RSLNM have not been evaluated using these imaging modalities. This study aimed to assess the prevalence and risk factors for RPLNM and RSLNM using a combination of MR, FDG–PET, and CT imaging in patients with HPC.

## Methods

### Patients

Between January 2011 and December 2021, 195 patients of pathologically diagnosed squamous cell carcinoma of hypopharynx underwent RT at our institution. Of these, 170 patients underwent pretreatment contrast-enhanced (CE) MR imaging within the 4 weeks and FDG–PET/CT imaging within the 6 weeks before CT imaging for RT planning. Of these, we excluded 15 patients because they had undergone RT and/or surgery before imaging or had coexisting esophageal, lung, and/or head and neck cancers at other subsites. After the exclusions, the final study population included 155 patients. Clinical staging was conducted by a head and neck tumor board of our hospital consisted of radiation oncologists, radiologists, and otolaryngologists. In addition to MR and FDG–PET/CT images, the staging was performed based on physical, ultrasound, and endoscopic examinations with or without fine-needle aspiration cytology specimens. We used 7th or 8th edition of Union for International Cancer Control TNM staging system.

### Assessment of pretreatment images

Details of the pretreatment imaging procedure have been described elsewhere [14]. CEMR images were acquired using a 3T-MR scanner. Fat-saturated eTHRIVE axial, sagittal, and coronal images with 1 mm slice intervals were acquired besides conventional axial images, including T1-weighted images (WI), T2WI, and short tau inversion recovery with 5 mm slice intervals [15, 16]. CE FDG–PET/CT images were acquired using a 3D PET/CT scanner. Dynamic CE scans were conducted to obtain 2 mm slice interval images. The FDG–PET/CT images of 4 mm slice intervals were also acquired [17–19]. CE or non-CE CT imaging for RT planning were performed to obtain 2.5 mm slice interval images. A thermoplastic mask and an RT pillow were used with each patient [19, 20].

Two board-certified radiation oncologists, who experienced 16 and 18 years in the diagnosis and treatment of head and neck cancers independently reviewed the CT and MR images, the FDG–PET and FDG–PET/CT-fused images, and the RT planning CT images. Observers assessed these images without prior knowledge of the patient’s clinical information and disagreements were resolved by consensus. The radiological diagnostic criteria for lateral RPLNM and RSLNM were a short-axis diameter of ≥ 5 mm and/or necrosis and/or abnormal FDG uptake [14, 21–23]. Any visible medial RPLN was defined as LNM [9, 10]. As diagnostic images were not obtained with the treatment position, correlations for level VII were made between the diagnostic and treatment position images. In addition to RSLNM assessments, including the measurement of the short-axis diameter and the maximum standardized uptake value (SUV_max_), the longest diameter of the largest LN in the upper limit of ipsilateral level II, which is located at the caudal edge of the C1 lateral process, was measured for each patient based on a previously reported risk factor for RSLNM in oropharyngeal carcinomas (OPC) [14].

### Statistical analysis

We performed statistical calculations using SPSS v.26.0 (IBM, Armonk, NY, USA) software. Fisher’s exact test was used to identify potential risk factors for RPLNM from the following variables: smoking status, tumor site, T category, histological grade, cervical LNM, and the maximum diameter of the largest ipsilateral LN. Factors with *p*-values of < 0.05 were subjected to logistic regression analysis. Moreover, Fisher’s exact test was performed to assess the relationship between the tumor site and contralateral RPLNM, and between the longest diameter of the largest LN in the upper limit of ipsilateral level II and RSLNM. Cut-off of ≥ 15 mm, which is the most commonly considered size criterion for LNM in level II, was used for the longest diameter of the largest LN in the upper limit of ipsilateral level II based on the previous report [14]. Differences with *p*-values < 0.05 were defined as statistically significant.

## Results

### Patient characteristics

Clinical N categories and other patient characteristics are summarized in Tables 1 and 2, respectively. There were 143 men and 12 women in our sample, with a median age of 69 years (range 38–91).

**Table 1:** Clinical N category classification of patients in this study based on the Union for International Cancer Control TNM staging system

UICC edition	*n*	N category (%)							
		N0	N1	N2a	N2b	N2c	N3	N3a	N3b
7th	77	32 (42)	8 (10)	1 (1)	23 (30)	12 (16)	1 (1)	NA	NA
8th	78	22 (28)	4 (5)	0 (0)	29 (37)	9 (12)	NA	0 (0)	14 (18)

UICC: Union for International Cancer Control; NA: not applicable

**Table 2:** Patient characteristics and Fisher’s exact test results for the identification of risk factors for retropharyngeal lymph node metastasis

Variables	Total cohort (Column %)	RPLN positive (Column %)	RPLN negative (Column %)	*p*-value
	*n* = 155	*n* = 20	*n* = 135	
Smoking status				
Never	13 (8)	2 (10)	11 (8)	0.877
Former	79 (51)	10 (50)	69 (51)	
Current	63 (41)	8 (40)	55 (41)	
Tumor site				
Pyriform sinus	110 (71)	10 (50)	100 (74)	0.006
Postcricoid region	17 (11)	1 (5)	16 (12)	
Posterior wall	28 (18)	9 (45)	19 (14)	
T category				
T1	17 (11)	0 (0)	17 (13)	0.049
T2	70 (45)	6 (30)	64 (47)	
T3	39 (25)	9 (45)	30 (22)	
T4	29 (19)	5 (25)	24 (18)	
Histological grade				
Well	27 (17)	1 (5)	26 (19)	0.065
Moderate	79 (51)	16 (80)	63 (47)	
Poor	22 (14)	1 (5)	21 (16)	
Not graded	27 (17)	2 (10)	25 (19)	
Cervical LNM				
No	56 (36)	2 (10)	54 (40)	< 0.001
Ipsilateral single	12 (8)	0 (0)	12 (9)	
Ipsilateral multiple	60 (39)	8 (40)	52 (39)	
Bilateral or contralateral	27 (17)	10 (50)	17 (13)	
Largest ipsilateral LN				
< 20 mm	107 (69)	10 (50)	97 (72)	0.112
20 to < 30 mm	28 (18)	6 (30)	22 (16)	
≥30 mm	20 (13)	4 (20)	16 (12)	

RPLN: retropharyngeal lymph node; LNM: lymph node metastasis; LN: lymph node

### Prevalence and factors associated with RPLNM

Of the 155 patients, 20 (13%) were diagnosed with RPLNM. Of these, 10 (6%) had ipsilateral RPLNM, 5 (3%) had contralateral RPLNM, and 5 (3%) had bilateral RPLNM. All RPLNM were solitary and located within level VIIa on the RT planning CT images. No patients were diagnosed with medial RPLNM. The median short-axis diameter and SUV_max_ of RPLNM were 13 mm (range 6–33 mm) and 9.1 (range, 1.7–22.1), respectively. Fisher’s exact test revealed tumor site (*p* = 0.006), T category (*p* = 0.049), and cervical LNM (*p* < 0.001) to be significantly associated with RPLNM (Table 2). Logistic regression analysis revealed posterior wall (PW) tumors (odds ratio [OR]: 4.128, 95% confidence interval [CI]: 1.339–12.727; *p* = 0.014) and bilateral or contralateral cervical LNM (OR: 11.577, 95% CI: 2.135–62.789; *p* = 0.005) to be significantly associated with RPLNM (Table 3).

**Table 3:** Logistic regression analysis results in the identification of risk factors for retropharyngeal lymph node metastasis

Variables	OR	95% CI	*p*-value
Tumor site			
Posterior wall	4.128	1.339–12.727	0.014
Others	1		
T category			
T1–2	1		
T3–4	2.206	0.702–6.932	0.175
Cervical LNM			
No/Ipsilateral single	1		
Ipsilateral multiple	4.190	0.796–22.066	0.091
Bilateral or contralateral	11.577	2.135–62.789	0.005

OR: odds ratio; CI: confidence interval; LNM lymph node metastasis

**Fig. 1:**
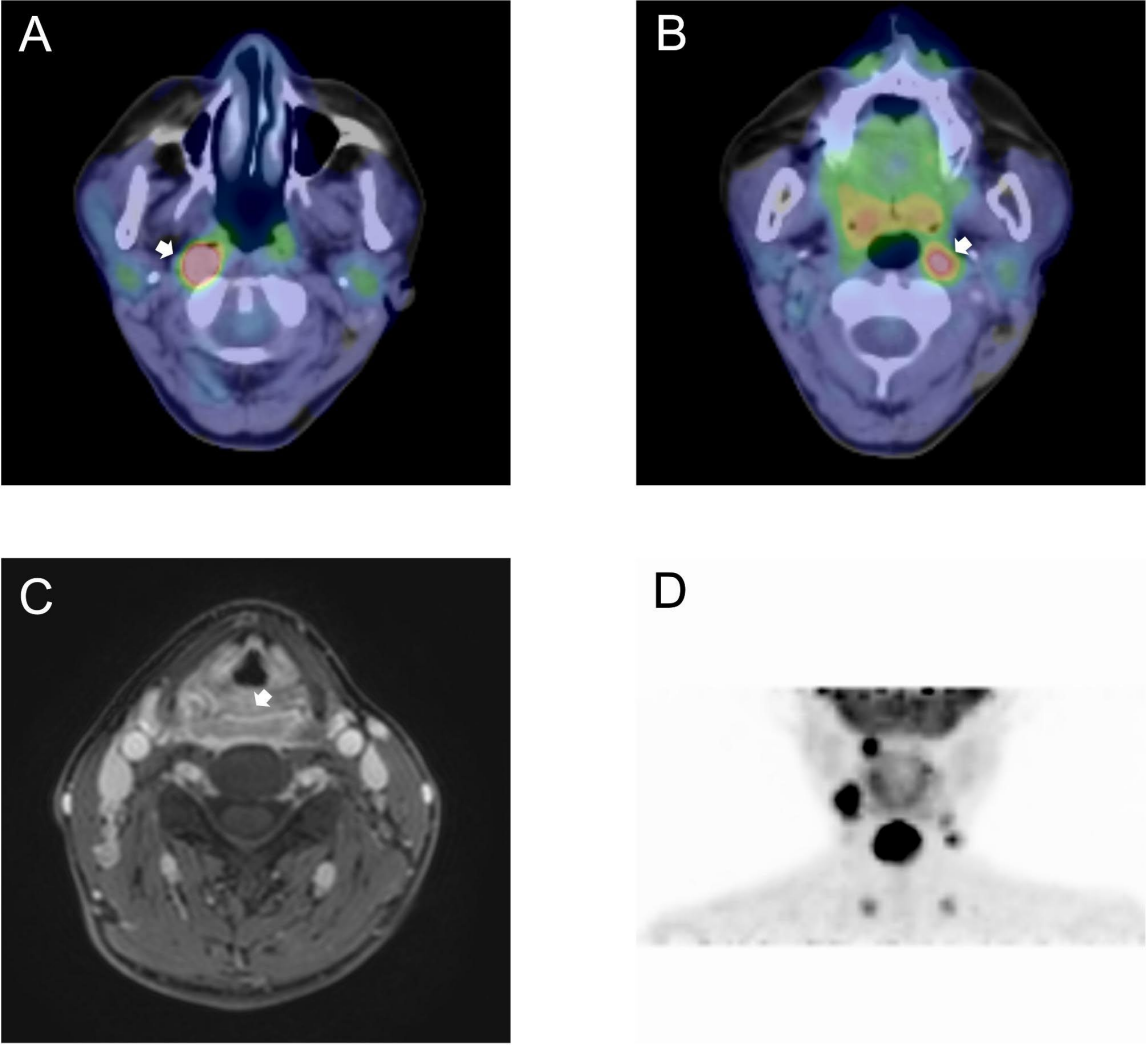
Hypopharyngeal carcinoma with bilateral retropharyngeal lymph node metastasis (RPLNM). **(A)** [^18^ F]-fluoro-2-deoxy-D-glucose (FDG)-positron emission tomography (PET)/computed tomography (CT)-fused images show right RPLNM (arrow; longest diameter = 14 mm, SUV_max_ = 11.4). **(B)** FDG–PET/CT-fused images show left RPLNM (arrow; longest diameter = 9 mm, SUV_max_ = 6.7). **(C)** Contrast-enhanced fat-saturated eTHRIVE images of the posterior wall primary tumor predominantly on the right side. **(D)** FDG–PET image revealed bilateral cervical LNM

Of the 28 patients with PW tumors, 9 (32%) had RPLNM. Of these 9 patients, 2 (7%) had ipsilateral RPLNM, 3 (11%) had contralateral RPLNM, and 4 (14%) had bilateral RPLNM. Of the remaining 127 patients, 11 (9%) had RPLNM. Among these 11, 8 (6%) had ipsilateral RPLNM, 2 (2%) had contralateral RPLNM, and 1 (1%) had bilateral RPLNM. The PW tumors were significantly associated with contralateral RPLNM (*p* < 0.001; Table 4; Fig. 1).

**Table 4:** Fisher’s exact test results for the identification of risk factors for contralateral retropharyngeal lymph node metastasis

Variables	Total cohort (Column %)	CRPLN positive (Column %)	CRPLN negative (Column %)	*p*-value
	*n* = 155	*n* = 10	*n* = 145	
Tumor site				
Posterior wall	28 (18)	7 (70)	21 (15)	< 0.001
Others	127 (82)	3 (30)	124 (86)	

CRPLN: contralateral retropharyngeal lymph node

### Prevalence and factors associated with RSLNM

**Fig. 2:**
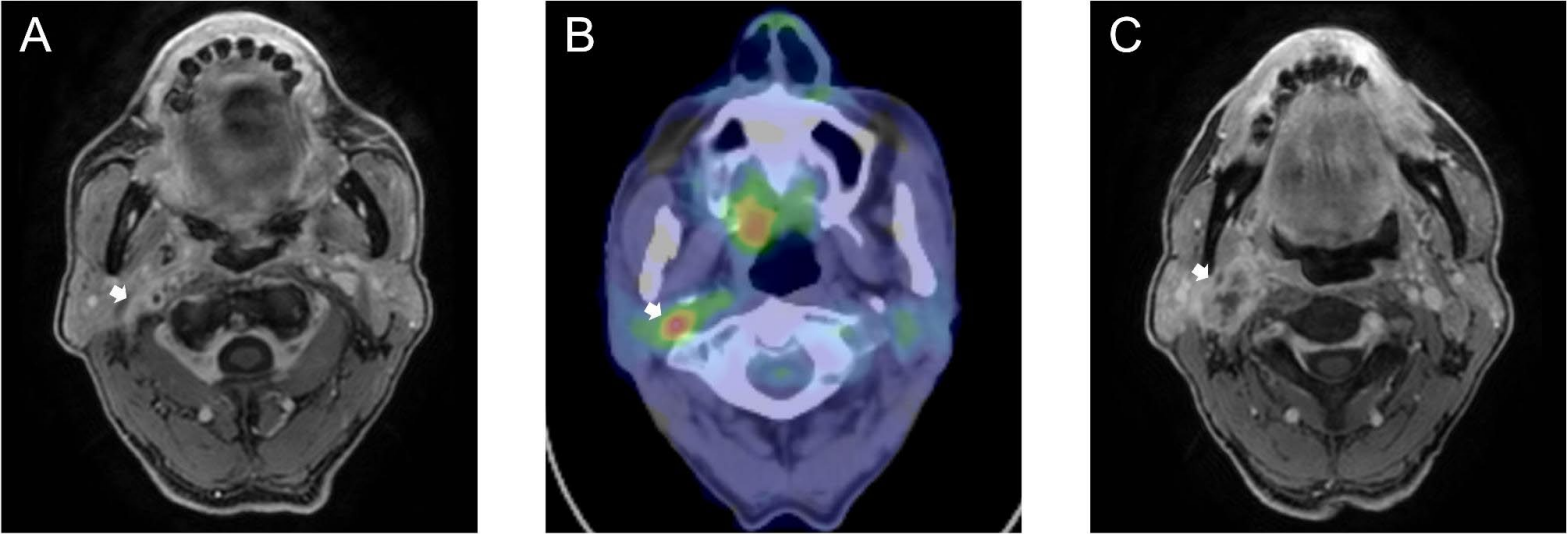
Hypopharyngeal carcinoma with ipsilateral retro-styloid lymph node metastasis (RSLNM). **(A)** Contrast-enhanced fat-saturated eTHRIVE and **(B)** [^18^ F]-fluoro-2-deoxy-D-glucose-positron emission tomography/computed tomography–fused images show RSLNM (arrow; longest diameter = 13 mm, SUV_max_ = 5.2). **(C)** Contrast-enhanced fat-saturated eTHRIVE images. The longest diameter of LN in the upper limit of ipsilateral level II was 25 mm (arrow)

Of the 155 patients, 2 (1%) were diagnosed with RSLNM. In both, the RSLNM was located within level VIIb on the RT planning CT images. The short-axis diameters and SUV_max_ of the two RSLNMs were 10 and 13 mm and 5.2 and 9.2, respectively. Both patients had ipsilateral RSLNM and LN of ≥ 15 mm in the upper limit of ipsilateral level II. The LN of ≥ 15 mm in the upper limit of ipsilateral level II was significantly associated with ipsilateral RSLNM (*p* = 0.001; Table 5; Fig. 2).

**Table 5:** Results of Fisher’s exact test for the identification of risk factors for retro-styloid lymph node metastasis

Variables	Total cohort (Column %)	RSLN positive (Column %)	RSLN negative (Column %)	*p*-value
	*n* = 155	*n* = 2	*n* = 153	
LN at the upper limit of ipsilateral level II				
< 15 mm	150 (97)	0 (0)	150 (98)	0.001
≥15 mm	5 (3)	2 (100)	3 (2)	

RSLN: retro-styloid lymph node; LN: lymph node

## Discussion

Table 6 summarizes studies of RPLNM diagnosed using different imaging modalities published between 2010 and the present time. The prevalence of RPLNM found in these studies was in the range of 10–20%. Therefore, the prevalence of 13% found in the present study was as per the previous research findings. However, the prevalence rates obtained may have been somewhat influenced by the backgrounds of the patients. This will be discussed further later.

**Table 6:** Comparison of research results from evaluations of retropharyngeal and retro-styloid lymph node metastases in hypopharyngeal carcinoma using different imaging modalities

Author	Year	N	Treatment	Imaging modalities	Prevalence rate of LNM		Risk factors of RPLNM
					Retropharyngeal	Retro-styloid	
Our study		155	(C)RT	MRI and FDG–PET/CT	13%	1%	PW tumor, bilateral or contralateral cervival LNM
An et al. [9]	2021	259	(C)RT	MRI	17%	NA	PW tumor, PW invasion, N2-3, multiple cervical LNM
Wang et al. [10]	2020	326	RT or preoperative RT (T3–4, N+)	MRI	22%	NA	PW tumor, bilateral LNM, GTVp, GTVn
Wu et al. [11]	2013	218	NA	CT or MRI	17%	NA	Tumor subsite, bilateral LNM, number of LNM, size of cervical LN, level V LNM
Deng et al. [12]	2010	88	NA	CT or MRI	14%	NA	NA
Chan et al. [13]	2010	122	CRT	FDG–PET	17%	NA	PW tumor, Ipsilateral level V LNM

LNM: lymph node metastasis; RPLNM: retropharyngeal lymph node metastasis; (C)RT: radiotherapy with or without chemotherapy; RT: radiotherapy; NA: not available; CRT: chemoradiotherapy; MRI: magnetic resonance imaging; FDG–PET: [^18^ F]-fluoro-2-deoxy-D-glucose–positron emission tomography; CT: computed tomography; PW: posterior wall; GTV: gross tumor volume

Medial RPLNs consist of 1–2 very small nodes and rarely occur in adults [4, 24]. Wu et al. reviewed the CT or MR images of 218 patients with HPC [11] and found RPLNM in 37 (17%) patients. All of these were categorized as lateral RPLNM, with no visible medial RPLN. Our study supported the notion that medial RPLNM is extremely rare. We concur with the current consensus guidelines that it is appropriate to exclude medial RPLN from clinical target volume (CTV) [4].

It has been suggested that PWs have first-echelon drainage to RPLNs [25]. Previous studies based on imaging modalities have also suggested that PW tumors and PW invasion are risk factors for RPLNM [9, 10, 13]. Our results were consistent with these previous studies. Furthermore, our findings suggest that PW tumors are a risk factor for contralateral RPLNM. The current guidelines for selecting LN target volumes recommend that bilateral level VIIa is included in the CTV for patients with PW tumors regardless of the N category [2]. Our results strongly support this recommendation.

There are many lymphatic channels, both afferent and efferent, between cervical LNs and RPLNs, other than those between the PW and the RPLN [25]. An et al. assessed the risk factors for RPLNM in HPC patients using the MR images from 259 patients according to the UICC TNM staging system (7th ed.) [9]. They found that the N2–3 category and multiple cervical LNM were risk factors for RPLNM. Wu et al. explored the risk factors associated with RPLNM in HPC based on the CT or MR images of 218 patients [11]. They reported that bilateral LNM, the number of LNM, and the size of cervical LNs were risk factors for RPLNM [11]. Our results also suggest that advanced N category with bilateral or contralateral cervical LNM is a risk factor for RPLNM. Wang et al. reported a prevalence rate of 22% for RPLNM based on the MR images of 326 HPC patients [10]. Their prevalence rate was relatively high compared to those found by other studies, including ours (Table 6). This may be because they included patients who underwent preoperative RT for locoregionally advanced diseases; the proportion of patients with N2b–N3 categories was 76.6%. Hence, the patients having a background of N category influence RPLNM prevalence.

To the best of our knowledge, the prevalence of RSLNM has not been assessed in patients with HPC (Table 6). This is probably because level VIIb has been defined in the consensus guidelines relatively recently [4]. The imaging-based classification by Som et al. published in 1999 defines the base of the skull as the upper boundary of level II; therefore, using their classifications, level II includes level VIIb of the current consensus guidelines by Grégoire et al. [26]. Our results suggest that the prevalence of RSLNM is very low and level VIIb need not be routinely included in the CTV for patients with HPC. As level VIIb is located near the pharynx, parotid gland, pterygoid muscles, and mastoid cells, irradiation to this level can considerably reduce a patient’s quality of life due to adverse effects, such as mucositis, xerostomia, trismus, and otitis media [14, 27]. From this point of view, it is preferable and appropriate to manage level II and level VIIb separately. Toya et al. assessed the prevalence and risk factors for RSLNM in 137 patients with OPC based on MR and FDG–PET/CT images [14]. They found that 18 (13%) of the patients in their sample had ipsilateral RSLNM and none had contralateral RSLNM. Of these 18 patients, 17 (12%) had LN of ≥ 15 mm in the upper limit of ipsilateral level II. Logistic regression analyses revealed the presence of LNs of ≥ 15 mm in the upper limit of ipsilateral level II to be significantly associated with ipsilateral RSLNM. In our HPC series, only five patients (3%) had LNs of ≥ 15 mm in the upper limit of ipsilateral level II. The differing prevalence of RSLNM between patients with HPC and OPC may be due to differences in the distribution of LNM. Previous studies based on surgical specimens have revealed that LNM in level II is more common in patients with OPC than those with HPC [28, 29]. As level VIIb is the cranial continuation of level II, the LNM status of level II significantly influences the prevalence of RSLNM [14]. Although RSLNM is very rare in patients with HPC, LNs of ≥ 15 mm in the upper limit of ipsilateral level II are a risk factor for ipsilateral RSLNM. Thus, ipsilateral level VIIb should be included in the CTV in HPC patients with this risk factor. The current consensus guidelines recommend the inclusion of level VIIb in CTV in patients with bulky involvement of the upper part of level II [2]. Our results provided a specific criterion for this recommendation.

Our study had some limitations. First, this was a retrospective study based on a relatively small number of patients. Second, there was no pathologic confirmation regarding the radiologically defined LN statuses. Third, a potential selection bias for RT exists and may have influenced the prevalence findings for RPLNM and RSLNM and their associated risk factors. Finally, we cannot comment on whether the existence of RPLNM and RSLNM influences the prognosis of patients with HPC.

## Conclusions

This study comprehensively evaluated the prevalence and risk factors for RPLNM and RSLNM in patients with HPC using a combination of MR and FDG–PET/CT imaging. The RPLNM was identified in 13% of the patients. The PW tumors and bilateral or contralateral cervical LNM were risk factors for RPLNM; particularly, PW tumors were a specific risk factor for contralateral RPLNM. The RSLNM was very rare; however, LNs of ≥ 15 mm in the upper limit of ipsilateral level II were found to be a risk factor for ipsilateral RSLNM.

## Data Availability

The data supporting the findings of this study are not publicly available as they were used under license. However, the data are available from the authors upon reasonable request and with permission from the Institutional Review Board of Kumamoto University Hospital.

## List of abbreviations

RT: Radiotherapy
HPC: Hypopharyngeal carcinoma
LN: Lymph node
CT: Computed tomography
RPLN: Retropharyngeal lymph node
RSLN: Retro-styloid lymph node
RPLNM: Retropharyngeal lymph node metastasis
RSLNM: Retro-styloid lymph node metastasis
MR: Magnetic resonance
FDG: [^18^ F]-fluoro-2-deoxy-D-glucose
PET: Positron emission tomography
CE: Contrast-enhanced
WI: Weighted image
SUV_max_: Maximum standardized uptake value
OPC: Oropharyngeal carcinoma
PW: Posterior wall
OR: Odds ratio
CI: Confidence interval
CTV: Clinical target volume
